# Early Outcomes of a Novel Collared Triple-Tapered Femoral System in Primary Total Hip Arthroplasty

**DOI:** 10.3390/medicina62050934

**Published:** 2026-05-11

**Authors:** Laith Bahlouli, Olivia Schaffer, Jacob Stoebner, Anna Cohen-Rosenblum, Vinay K. Aggarwal, Ran Schwarzkopf

**Affiliations:** Department of Orthopedic Surgery, NYU Langone Health, New York, NY 10016, USA; bahlouli.laith@gmail.com (L.B.);

**Keywords:** triple-tapered femoral stem, collared femoral stem, patient-reported outcomes, primary total hip arthroplasty

## Abstract

*Background and Objectives*: Collared, triple-tapered femoral stems have gained increasing popularity in primary total hip arthroplasty (THA) due to their stable metaphyseal fixation and ability to restore native hip biomechanics. This study evaluated the short-term clinical and functional outcomes of a novel collared triple-tapered femoral stem design in primary THA. *Materials and Methods*: This was a retrospective review of all patients who underwent primary, elective THA using a collared, triple-tapered femoral system at a single, urban, high-volume, academic hospital between September 2024 and February 2025. All procedures were performed by fellowship-trained arthroplasty surgeons. A total of 101 patients (102 hips) with a median follow-up of 1.1 years (range, 1.0 to 1.4 years) were included. *Results*: Most procedures were performed for primary osteoarthritis (96%). Mean operative time, from skin incision to skin closure, was 93 min, and most femoral stems implanted had a high offset (89%). Most patients were discharged home (96%), with a mean length of stay of 27 h. Within 90 days, three patients were readmitted for surgery-related reasons: one for superficial wound dehiscence, and two for periprosthetic joint infection (PJI). One PJI was treated with irrigation and debridement, antibiotics, and implant retention (DAIR) two months after primary THA. The other required a DAIR three weeks after primary THA, followed by a single-stage revision one week later. No dislocations, periprosthetic fractures, mechanical failures, or aseptic revisions of the femoral stem occurred. All stems were well-fixed at the latest follow-up, with no aseptic loosening observed. Mean Hip disability and Osteoarthritis Outcome Score, Joint Replacement (HOOS, JR) improvement was 15.0 points at six weeks, 25.2 points at three months, and 45.3 points at one year. *Conclusions*: Our results support encouraging early outcomes with no femoral aseptic complications observed using this novel collared, triple-tapered femoral system. A longer follow-up period is needed to assess mid- and long-term durability.

## 1. Introduction

For patients with end-stage hip osteoarthritis, total hip arthroplasty (THA) is a highly effective orthopedic procedure consistently providing increased quality of life through substantial pain relief and functional improvement [1,2,3]. The demand for THA has increased substantially over recent decades, driven by an aging population, growing prevalence of osteoarthritis, and greater utilization among younger, more active patients [4,5]. More than 300,000 primary THAs are performed annually in the United States alone, and this volume is projected to increase substantially in the coming years [6,7]. The growing utilization of THA has important economic implications, as it remains one of the most commonly reimbursed procedures by Medicare and Medicaid, with total annual costs estimated in the billions of dollars [3]. As the prevalence of THA continues to rise, optimizing implant selection and surgical technique will be essential to minimizing complications and reducing the burden of revision THA on both patients and the wider healthcare system [7,8].

Despite THA’s success in the United States and worldwide, failures still occur due to early periprosthetic fracture (PPF) and aseptic loosening [9,10]. PPF is among the most serious adverse events following THA and may occur intraoperatively during stem implantation or postoperatively as a result of trauma, poor bone quality, or implant loosening [11,12]. Aseptic loosening is one of the most common indications for revision THA and occurs when fixation between the implant and surrounding bone fails in the absence of infection, often because of osteolysis, inadequate initial fixation, or progressive loss of fixation over time [13,14]. Although femoral stem design is primarily intended to optimize fixation and reduce mechanical complications, early evaluation of implants should also account for adverse events like periprosthetic joint infection (PJI), which can lead to readmission, reoperation, and substantial healthcare utilization after THA. PJI accounts for approximately 9–15% of all revision surgeries following primary THA, with the majority of infections presenting within the first two years and a disproportionate share occurring in the early postoperative period (within 90 days) [15,16]. Taken together, the occurrence of PPF, aseptic loosening, and PJI underscores the need for implant designs that enhance initial stability while preserving durable long-term fixation and maintaining a favorable early safety profile [17].

Cementless femoral stems are widely used in primary THA in North America [18]. However, cementless stems remain at risk for early PPF as well as proximal bone loss due to stress shielding, which can lead to subsidence and early failure [19,20,21,22]. Recent implant innovations, including collared triple-tapered stems designed for metaphyseal fixation, reduce these complications by loading the calcar, minimizing stress shielding and preserving femoral bone stock [23]. Calcar collars may also decrease torsional stresses on the femur, which are responsible for spiral fracture patterns observed in early PPFs prior to osseous integration [24]. Indeed, triple-tapered collared stems have displayed a low incidence of intra- and postoperative PPF compared to other cementless stem designs, and a comparable PPF incidence to cemented stem designs [25,26].

However, there are limited independent data on the early performance of modern collared triple-tapered femoral stems, particularly with respect to patient-reported outcomes and short-term complication profiles [27]. Early clinical evaluation of novel implant designs is important for identifying potential modes of failure and guiding surgeon decision-making before widespread implementation. This study evaluated the short-term clinical and functional outcomes of a novel femoral stem system featuring a collared triple-tapered design in primary THA.

## 2. Materials and Methods

### 2.1. Study Design

A retrospective review was conducted of all patients who underwent unilateral, primary, elective THA using a novel collared triple-tapered femoral system (CATALYSTEM; Smith & Nephew, Memphis, TN, USA). Patients younger than 18 years of age, missing operative information, or had follow-up of less than one year were excluded. All procedures were performed at an urban, high-volume, academic hospital between September 2024 and February 2025 by six fellowship-trained arthroplasty surgeons. Institutional review board (IRB) approval was obtained before initiating this study: i17-01223. The implant manufacturer had no role in the study design, data collection, or data analysis.

Perioperative management followed institutional protocols during the study period. An opioid-sparing multimodal pain regimen was used, consisting of preoperative and postoperative oral meloxicam and acetaminophen, with tramadol or low-dose oxycodone available as needed for breakthrough pain. Anesthesia was predominantly spinal, with general anesthesia used in a minority of cases. Chlorhexidine-based skin preparation was utilized. Perioperative antibiotic prophylaxis consisted of cefazolin with additional Gram-negative coverage as standard therapy. In patients colonized with methicillin-resistant *Staphylococcus aureus* (MRSA) or in patients with anaphylactic cephalosporin allergy, cefazolin was replaced with weight-based vancomycin. Gram-negative coverage consisted primarily of gentamicin, with aztreonam used when indicated. Following implantation, wounds were irrigated with dilute povidone-iodine (0.35%) for approximately three minutes, followed by copious saline irrigation. Topical vancomycin powder (2 g) was applied prior to closure. Venous thromboembolism (VTE) prophylaxis was risk-stratified, with aspirin 81 mg twice daily used for standard-risk patients and stronger anticoagulation, such as enoxaparin, reserved for patients with prior VTE, active malignancy, and other risk factors. Intermittent pneumatic compression devices were used during the inpatient stay. Blood-loss prevention included routine administration of tranexamic acid (TXA), most commonly by the intravenous route (1 g before incision and 1 g at closure), while topical TXA was used when intravenous dosing was contraindicated.

### 2.2. Data Collection

Data were collected through a comprehensive chart review of our institution’s electronic medical record (Epic Caboodle, Version 15; Verona, WI, USA). Baseline patient characteristics included age, body mass index (BMI), sex, race, smoking status, diabetes status, American Society of Anesthesiologists (ASA) class, and Charlson Comorbidity Index (CCI). Perioperative data included surgical indication, laterality, anesthesia type, surgical approach, operative time, length of stay, and discharge disposition. Implant characteristics captured were femoral stem size and offset, femoral head diameter and neck-length, acetabular cup diameter, and whether a dual mobility construct was used.

Postoperative complications included surgery-related 90-day emergency department visits and readmissions, in addition to revisions through to the latest follow-up. Latest follow-up radiographs for each hip were reviewed by the senior author for evidence of radiographic loosening. Functional outcomes were measured preoperatively and at six weeks, three months, and one year postoperatively using the patient-reported Hip disability and Osteoarthritis Outcome Score, Joint Replacement (HOOS, JR). Postoperative HOOS, JR at each time point was reported based on available follow-up data, and improvements in HOOS, JR were calculated using paired preoperative and postoperative observations.

Categorical and ordinal variables were summarized as counts and percentages, while continuous variables were summarized as means and standard deviations or medians and ranges. Individual hips were used as the unit of analysis for all reported characteristics and outcomes.

### 2.3. Baseline Characteristics

The final cohort comprised 101 patients (102 hips), with one patient undergoing a staged bilateral procedure three months apart. Median age was 65 years (range, 26 to 79 years), median BMI was 29 kg/m^2^ (range, 15 to 48 kg/m^2^), and median follow-up was 1.1 years (range, 1.0 to 1.4 years). The cohort was evenly divided by sex, with 51 hips in men (50%) and 51 in women (50%). Most hips were in White patients (63%), followed by Black (21%) and Asian patients (5%); 12% were in patients who identified with another race. Just over half of the hips were in patients who had never smoked (57%), whereas 38% were in former smokers and 5% were in current smokers. Eighteen patients had diabetes. Most hips were in patients with an ASA class of II (74%), while 23% were in ASA class III patients and 4% in ASA class I patients. The cohort mean CCI was 1.5 ± 2.1 (Table 1).

## 3. Results

### 3.1. Perioperative Characteristics

Most procedures were performed for primary osteoarthritis (96%), with three cases for avascular necrosis, and one for adult hip dysplasia. Fifty-five procedures (54%) involved the right hip, and 47 (46%) involved the left. Spinal anesthesia was used in nearly all cases (94%), while general anesthesia was used in six (6%). The superior transverse anatomic reconstruction (STAR) approach was utilized in the majority of cases (75%), while twenty-six procedures were performed through a traditional posterior approach. Mean operative time, defined as the average time from skin incision to skin closure, was 93 ± 20 min. Most procedures resulted in discharge to home (96%), whereas four (4%) resulted in discharge to a skilled nursing facility, following a mean length of stay of 27 ± 30 h (Table 2).

### 3.2. Implant Characteristics

Most femoral stems implanted had a high offset (89%), while 11% had a standard offset, with a median size of 5 (range, 0 to 9). The median femoral head diameter was 36 mm (range, 32 to 44 mm), and the median femoral head neck-length was 4 mm (range, −3 to 8 mm). The median acetabular cup diameter was 52 mm (range, 46 to 62 mm), and nine cases (9%) involved dual-mobility constructs (Table 3).

### 3.3. Postoperative Complications

**Figure 1:**
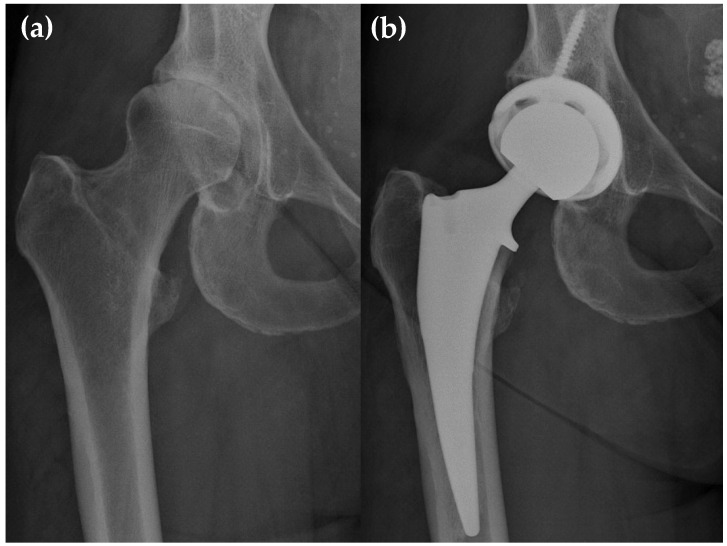
Representative case of primary THA using a collared, triple-tapered femoral stem: (**a**) Preoperative radiograph of the right hip showing end-stage primary osteoarthritis; (**b**) one-year postoperative radiograph demonstrating appropriate implant positioning and stable fixation without evidence of subsidence, loosening, or periprosthetic fracture.

Within 90 days, three patients were readmitted for surgery-related reasons: one for superficial wound dehiscence, admitted directly from the emergency department, and two for PJI. One PJI was treated with irrigation and debridement, antibiotics, and implant retention (DAIR) two months after primary THA. The other required a DAIR three weeks after primary THA, followed by a single-stage revision one week later. No dislocations, periprosthetic fractures, mechanical failures, or aseptic revisions of the femoral stem occurred (Table 4). All stems were well-fixed at the latest follow-up, with no aseptic loosening observed. Representative radiographs are shown below in Figure 1.

Four additional 90-day encounters, unrelated to primary THA, occurred and were excluded from surgery-related complication reporting. These included one readmission each for syncope, influenza, and spinal fusion surgery, as well as an emergency department visit for pneumonia that resulted in hospital admission.

### 3.4. Functional Outcomes

Among the 102 hips, mean HOOS, JR improvement was 15.0 ± 19.1 points at six weeks (n = 33), 25.2 ± 12.5 points at three months (n = 36), and 45.3 ± 18.7 points at one year (n = 53) (Table 5).

## 4. Discussion

This study evaluated early clinical and functional outcomes following primary total hip arthroplasty using a novel collared, triple-tapered femoral stem system with favorable one-year results. Most patients were discharged home after an average hospital stay of 27 h, complications were uncommon, and no dislocations, periprosthetic fractures, or revisions of the femoral stem for mechanical failure or aseptic loosening were observed during the follow-up period. Patients also experienced progressive improvement in HOOS, JR, with mean improvements of 15.0 points at six weeks, 25.2 points at three months, and 45.3 points at one year. These findings suggest that patients derived functional benefit from the procedure in the early postoperative period.

Long-term studies have demonstrated excellent survivorship of triple-tapered femoral stems with a low incidence of aseptic loosening. Tyrpenou et al. reported on 376 consecutive THAs using a cementless triple-tapered stem and found 97.7% survivorship at 15–19 years, with no revisions for aseptic loosening [28]. Carlson et al. similarly reported 100% survivorship at a minimum of 10 years in a series of 100 THAs using a contemporary triple-tapered titanium stem, with no femoral revisions for loosening and no radiographic loosening observed [29]. Even longer-term data support the durability of tapered titanium stems, with 87% femoral component survivorship and 94% survivorship for aseptic loosening at 28 years [30]. Although our follow-up was limited to one year, the absence of aseptic revisions in this cohort is consistent with the low incidence of femoral failure reported with triple-tapered designs.

The collared design of the femoral stem evaluated in this study may also contribute to the favorable complication profile observed. Hamoudi et al. analyzed femoral stems from more than 52,000 THAs and found a lower cumulative incidence of all-cause revision in the collared uncemented group than in the collarless uncemented and cemented groups, with the lowest revision incidence for periprosthetic fracture [31]. Favroul et al. also found that the presence of a collar was the only independent factor associated with reduced early periprosthetic fracture risk, and Ricotti et al. demonstrated that transitioning from collarless to collared triple-tapered femoral components reduced periprosthetic fracture incidence from 1.42% to 0.13% [32,33]. Biomechanical studies have also provided insight into the mechanism underlying this protective effect: Brand et al. demonstrated that collared stems tolerate substantially greater axial load before failure and alter strain distribution along the proximal femur, increasing compressive forces while reducing tensile hoop stresses that predispose to fracture [34]. The absence of postoperative periprosthetic fractures in the present series is consistent with these prior findings.

The geometry of the stem used in this study reflects a design aimed at preserving proximal femoral bone stock while maintaining reliable metaphyseal fixation, resulting in comparable fixation to longer stems. In a prospective multicenter registry series of 5876 short femoral stems, Delaunay et al. reported a cumulative revision incidence of 1.4% at approximately 10 years [35]. Similarly, analysis of the Dutch Arthroplasty Register by Van Veghel et al. found comparable revision incidence between commonly used short stems and standard-length stems [36]. Randomized evidence also supports similar clinical performance, with Won et al. reporting no meaningful differences in thigh pain, bone loss, or functional outcomes between short and conventional stems at five years following THA [37].

The incidence of PJI observed in this cohort falls within the range reported in the contemporary literature. Triantafyllopoulos et al. evaluated 36,494 primary THAs and reported a deep infection incidence of approximately 0.4%, with early infections occurring in roughly 0.3% of cases [38]. Population-based studies have demonstrated cumulative infection incidences of approximately 0.5–0.6% at 90 days and 1.4–1.5% over longer follow-up periods [16,39,40]. Registry data also suggests that revisions for infection have become more common in recent years, with much of this risk concentrated in the early postoperative period. Daleet al., analyzing data from the Nordic Arthroplasty Register Association, found that the hazard ratio for revision due to infection increased in more recent surgical eras, with most infections presenting within the first 90 days after surgery [41]. Although the present study includes approximately one year of follow-up and captures the period when many infections arise, continued surveillance is warranted to ensure that no late infections emerge over time, which may occur due to delayed presentation or biofilm accumulation.

Functional outcomes in this cohort improved substantially over the first postoperative year, with a mean HOOS, JR improvement of 45.3 points at one year. HOOS, JR is a widely used and validated measure for assessing pain, function, and overall patient-reported success following total hip arthroplasty [42]. Prior studies have demonstrated that acceptable improvements in HOOS, JR may vary based on factors such as underlying diagnosis, BMI, and other patient characteristics [43,44,45]. Despite this variability, the magnitude of improvement observed in the present cohort is encouraging and suggests that patients experienced meaningful functional recovery following surgery.

These findings should be interpreted in the context of several study limitations. This study did not include a control group using an alternative femoral stem design, limiting the ability to directly compare outcomes between implants. Follow-up was limited to one year, which restricts assessment of mid- and long-term survivorship and complications such as aseptic loosening. Additionally, radiographic assessment was based on the senior author’s review of the latest available follow-up imaging rather than a standardized radiographic analysis, which may limit the rigor of reported aseptic loosening findings. Patient-reported outcome follow-up was also incomplete at several postoperative timepoints; thus, HOOS, JR analyses based on available follow-up may have been subject to response bias. This study was conducted at a single urban, high-volume academic orthopedic center, which may limit generalizability to other practice environments with different patient populations or surgical volumes. The cohort only included four non-OA patients and did not include patients undergoing THA for hip fracture or inflammatory arthritis, nor did it include patients aged 80 or above; therefore, the findings may be difficult to extrapolate to all patients. There were no anterior-approach THAs in this cohort, which has become an increasingly popular approach among arthroplasty surgeons [46].

## 5. Conclusions

This study supports favorable early clinical outcomes and meaningful functional improvement following primary THA using a novel collared triple-tapered femoral system. Continued follow-up will be necessary to determine whether these encouraging early findings translate into durable mid- and long-term implant performance.

## Figures and Tables

**Table 1:** Baseline characteristics.

Parameter	n = 102
Age (years), median [range]	65 [26–79]
BMI (kg/m^2^), median [range]	29 [15–48]
Men, n (%)	51 (50)
Race, n (%)	
White	64 (63)
Black	21 (21)
Asian	5 (5)
Other	12 (12)
Smoking Status, n (%)	
Current	5 (5)
Former	39 (38)
Never	58 (57)
Diabetes, n (%)	18 (18)
ASA Class, n (%)	
I	4 (4)
II	75 (74)
III	23 (23)
CCI, mean [SD]	1.5 [2.1]
Follow-up (years), median [range]	1.1 [1.0–1.4]

BMI, Body mass index; kg, kilogram; m, meter; n, number of; %, percent; ASA, American Society of Anesthesiologists; CCI, Charlson Comorbidity Index; SD, standard deviation.

**Table 2:** Perioperative characteristics.

Parameter	n = 102
Surgical Indication, n (%)	
Primary osteoarthritis	98 (96)
Avascular necrosis of femoral head	3 (3)
Adult hip dysplasia	1 (1)
Right Hip, n (%)	55 (54)
Anesthesia Type, n (%)	
Spinal	96 (94)
General	6 (6)
Surgical Approach, n (%)	
Superior Transverse Anatomic Reconstruction (STAR)	76 (75)
Posterior	26 (25)
Operative Time (minutes), mean [SD]	93 [20]
Length of Stay (hours), mean [SD]	27 [30]
Discharge Disposition, n (%)	
Home	98 (96)
Skilled Nursing Facility	4 (4)

n, Number of; %, percent; SD, standard deviation.

**Table 3:** Implant characteristics.

Parameter	n = 102
Femoral Stem Size, median [range]	5 [0–9]
Femoral Stem Offset, n (%)	
Standard	11 (11)
High	91 (89)
Femoral Head Diameter (mm), median [range]	36 [32–44]
Femoral Head Neck-Length (mm), median [range]	4 [−3–8]
Dual Mobility, n (%)	9 (9)
Acetabular Cup Diameter (mm), median [range]	52 [46–62]

n, Number of; %, percent; mm, millimeter.

**Table 4:** Postoperative complications.

Parameter, n	n = 102
90-day Emergency Department Visit	1
90-day Readmission	3
Septic Revision	2
Debridement, Antibiotics, and Implant Retention	1
Single-Stage	1
Aseptic Revision	0

n, Number of.

**Table 5:** Hip disability and Osteoarthritis Outcome Score, Joint Replacement (HOOS, JR).

Parameter, mean [SD], n	n = 102
Preoperative	45.4 [15.4], 93
At 6 weeks	59.8 [13.7], 35
At 3 months	71.6 [17.1], 38
At 1 year	89.0 [16.9], 60
∆ 6 weeks	15.0 [19.1], 33
∆ 3 months	25.2 [12.5], 36
∆ 1 year	45.3 [18.7], 53

SD, Standard deviation; n, number of.

## Data Availability

The original contributions presented in this study are included in the article. Further inquiries can be directed to the corresponding author.

## Abbreviations

The following abbreviations are used in this manuscript:

THA	Total hip arthroplasty
PPF	Periprosthetic fracture
BMI	Body mass index
ASA	American Society of Anesthesiologists
CCI	Charlson Comorbidity Index
HOOS, JR	Hip disability and Osteoarthritis Outcome Score, Joint Replacement
PJI	Periprosthetic joint infection
DAIR	Debridement, antibiotics, and implant retention
